# Curcumol inhibits ferritinophagy to restrain hepatocyte senescence through YAP/NCOA4 in non‐alcoholic fatty liver disease

**DOI:** 10.1111/cpr.13107

**Published:** 2021-08-03

**Authors:** Xiaoyu Qi, Anping Song, Mingyue Ma, Peipei Wang, Xinbei Zhang, Chunfeng Lu, Junxiu Zhang, Shuguo Zheng, Huanhuan Jin

**Affiliations:** ^1^ Department of Pharmacology School of Pharmacy Wannan Medical College Wuhu China; ^2^ School of Pharmacy Nantong University Nantong China

**Keywords:** cellular senescence, curcumol, ferritinophagy, iron overload, non‐alcoholic fatty liver disease, nuclear receptor coactivator 4, Yes‐associated protein

## Abstract

**Objectives:**

In recent years, cellular senescence has attracted a lot of interest in researchers due to its involvement in non‐alcoholic fatty liver disease (NAFLD). However, the mechanism of cellular senescence is not clear. The purpose of this study was to investigate the effect of curcumol on hepatocyte senescence in NAFLD and the molecular mechanisms implicated.

**Materials and methods:**

LVG Golden Syrian hamsters, C57BL/6J mice and human hepatocyte cell line LO2 were used. Cellular senescence was assessed by analyses of senescence marker SA‐β‐gal, p16 and p21, H3K9me3, γ‐H2AX and telomerase activity.

**Results:**

The results showed that curcumol could inhibit hepatocyte senescence in both in vivo and in vitro NAFLD models, and the mechanism might be related to its regulation of ferritinophagy and subsequent alleviation of iron overload. Moreover, overexpression of nuclear receptor coactivator 4 (NCOA4) weakened the effect of curcumol on ferritinophagy‐mediated iron overload and cellular senescence. Furthermore, we demonstrated that curcumol reduced the expression of NCOA4 by Yes‐associated protein (YAP). In addition, depression of YAP could impair the effect of curcumol on iron overload and cellular senescence.

**Conclusion:**

Our results clarified the mechanism of curcumol inhibition of hepatocyte senescence through YAP/NCOA4 regulation of ferritinophagy in NAFLD. These findings provided a promising option of curcumol to regulate cellular senescence by target YAP/NCOA4 for the treatment of NAFLD.

## Graphical abstract

Curcumol inhibited hepatocyte senescence by suppressing ferritinophagy‐mediated iron overload. Furthermore, this effect of curcumol is related to the regulation of YAP/NCOA4 in NAFLD models.

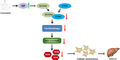

## 1 INTRODUCTION

Non‐alcoholic fatty liver disease (NAFLD), a manifestation of metabolic syndrome, affects 20%‐30% of the population in the worldwide scale.^1^, ^2^ NAFLD covers a wide spectrum of disorders ranging from hepatic steatosis to non‐alcoholic steatohepatitis, liver fibrosis, cirrhosis and even hepatocellular carcinoma.^3^ The hallmark of NAFLD is excessive intracellular accumulation of triglycerides in hepatocytes due to the imbalance between lipid deposition and clearance.^4^ Previous studies have shown that hepatocyte senescence exists in the liver of NAFLD patients and aggravates liver steatosis. However, inhibition of hepatocyte senescence could improve the pathological process of NAFLD.^5^, ^6^ Cellular senescence is a phenomenon in which the physiological function of cells is attenuated, including decreased proliferation capacity, cell cycle withdrawal and increased expression of ageing‐related genes.^7^ However, there are few studies focused on the senescence of hepatocytes in NAFLD, and little is known about how to regulate the cellular senescence to improve NAFLD.

Iron, one of the important trace elements in human, participates in the transportation of oxygen in the body, maintains normal hematopoietic function and immune function.^8^ Excessive deposition of iron would cause pathological iron overload.^9^ Growing evidence has shown that pathological iron overload in chronic liver disease is highly correlated with the activation of ferritinophagy,^10^, ^11^ which mainly consists of two parts, the formation of autophagosomes and the targeted recognition of ferritin.^12^ Nuclear receptor coactivator 4 (NCOA4) is one of the specific mediators of ferritinophagy to selectively degrade ferritin.^13^, ^14^ NCOA4 is highly enriched in autophagosomes and interacts with ferritin heavy chain 1 (FTH1), which mediates the target recognition of FTH1 by autophagosomes.^15^ In addition, iron could catalyse the generation of reactive oxygen species (ROS) through Fenton reaction and promote lipid peroxidation, which would lead to further oxidative damage to cells^16^ including cellular senescence.^17^ In short, it is quite interesting to explore whether NCOA4 influences hepatocyte senescence through ferritinophagy‐mediated pathological iron overload in NAFLD.

Yes‐associated protein (YAP), a key regulator of the Hippo signalling pathway, could affect the formation of autophagosomes,^18^ which is an important part of ferritinophagy.^14^ In addition, YAP could regulate cellular senescence.^19^ Thus, whether YAP regulates cellular senescence through inhibition of ferritinophagy and subsequent pathological iron overload is worth further exploring.

Curcumol is an ingredient extracted from the volatile oil of a traditional Chinese herb zedoary turmeric.^20^ Evidence has supported its efficacy in protection against liver fibrosis and hepatocellular carcinoma.^21^, ^22^, ^23^ However, the effects of curcumol on NAFLD have not yet been reported. Our preliminary researches have shown that curcumol could increase the expression of YAP, reduce iron content and inhibit cellular senescence in PA‐treated hepatocyte. But whether the ameliorative effect of curcumol on hepatocyte senescence is related to its regulation on YAP and cellular iron content remains unclear. The objective of this study was to evaluate the effects of curcumol on hepatocyte senescence and to elucidate the possible mechanisms implicated from the aspect of iron overload based on the YAP/NCOA4 signalling pathway.

## 2 MATERIALS AND METHODS

### 2.1 Reagents and antibodies

The following compounds and reagents were used in this study: curcumol (Herbpurify); palmitic acid (PA) (Solarbio); ferric ammonium citrate (FAC) (Solarbio); senescence‐associated β‐galactosidase (SA‐β‐gal) staining kit (Cell Signaling Technology); Oil Red O (Sigma); Reactive Oxygen Species (ROS) Assay Kit (Beyotime); Antifade Mounting Medium (Beyotime); DMEM (GIBCO BRL); foetal bovine serum (ExCell Bio); alanine aminotransferase (ALT), aspartate transaminase (AST), alkaline phosphatase (ALP), total cholesterol (T‐CHO), triglyceride (TG), low‐density lipoprotein cholesterol (LDL‐C) and high‐density lipoprotein cholesterol (HDL‐C) (Nanjing Jiancheng Bioengineering Institute); Tissue Iron Content Colorimetric Assay Kit (Nanjing Jiancheng Bioengineering Institute); Iron Colorimetric Assay Kit (APPLYGEN); Lipofectamine2000 reagent, TRIzol reagent (Invitrogen^TM^, Thermo Fisher Scientific); RevertAid First Strand cDNA Synthesis Kit, Pierce Co‐Immunoprecipitation (Co‐IP) Kit (Thermo Scientific™, Thermo Fisher Scientific); PowerUp™ SYBR® Green Master Mix (Applied Biosystems™, Thermo Fisher Scientific); NCOA4 CRISPR Activation Plasmid, UltraCruz® Transfection Reagent (Santa Cruz Biotechnology); YAP shRNA (GenePharma). The following primary antibodies were used in this study: YAP, p16, p21, H3K9me3, γ‐H2AX (Cell Signaling Technology); NCOA4, TRF2, Atg1, p62, Beclin1, FTH1 (Santa Cruz Biotechnology); TERT, TRF1 (Bioss); LC3, β‐actin (Proteintech Group).

### 2.2 Experimental animal procedures

All experimental procedures were allowed by the institutional and local committee on the care and use of animals of Wannan Medical College (Wuhu, China) and all animals received humane care according to the National Institutes of Health (USA) guidelines. LVG Golden Syrian hamsters (80–110 g body weight) were bought from Beijing Vital River Laboratory Animal Technology Co., Ltd. High fat food was used to induce non‐alcoholic fatty liver in animals. A total of 40 hamsters were randomly divided into five groups (n = 8) following adaptive feeding for 1 week. Group 1 was the vehicle control in which hamsters were given a normal chow diet (NCD). Animals in group 2‐5 were given a high fat diet (HFD) for 12 weeks, while those in group 3‐5 were additionally given curcumol (15, 30 and 60 mg/kg) by gavage from week 9 to week 12. At the end of experiments, all hamsters were anaesthetized with pentobarbital and blood was collected from the abdominal aorta. Then, animals were sacrificed and liver was isolated. Part of the liver was fixed in 10% neutral buffered formalin for histological analysis, and the rest was stored at −80°C for further analyses.

Male C57BL/6J mice of 8 weeks (20–22 g body weight) were obtained from Henan Skbex Biotechnology Co., Ltd. Model Diet of Non‐alcoholic fatty liver disease (TP26300) in C57BL/6J mice were purchased from Nantong Trophic Biotechnology Co., Ltd. After 1 week of adaptive feeding, 40 animals were randomly divided into five groups (n = 8): (a) NCD + control group, (b) NCD + empty vector group, (c) HFD + empty vector group, (d) HFD + empty vector +curcumol (30 mg/kg) group and (e) HFD +NCOA4 plasmid +curcumol (30 mg/kg) group. Mice in groups (c)‐(e) were given model diet for 12 weeks. Curcumol were suspended in sterile phosphate‐buffered saline (PBS) and given daily by gavage during weeks 9‐12. After 8 weeks, mice in groups (b), (c) and (d) were injected with empty vector and mice in group (e) were injected with NCOA4 plasmid through the tail veil. The lentivirus‐mediated NCOA4 plasmid and empty vector were designed and synthesized by GenePharma. Lentivirus vector (1 × 10^9^ TU/mL, once per 10 days) were injected into mice for 30 days. At the end of experiment, animals were anaesthetized with pentobarbital and blood samples and liver tissues were stored at −80°C for future experiments.

### 2.3 Cell culture

The human immortalized normal hepatocyte cell line LO2 was bought from Cell Bank of Chinese Academy of Sciences and cultured in DMEM added with 10% foetal bovine serum, 1% penicillin and streptomycin (PS) at 37°C in a humidified atmosphere containing 5% CO_2_.

### 2.4 Serum biochemical analysis

The serum was taken after centrifugation of whole blood, and the levels of ALT, AST, ALP, T‐CHO, TG, LDL‐C and HDL‐C were detected by related assay kit according to the manufacturer's protocol.

### 2.5 Haematoxylin and eosin staining

The liver fixed in 10% formalin solution was embedded routinely into paraffin and cut into 4 μm slices. After deparaffinization and hydration, the sections were subjected to haematoxylin and eosin (H&E) staining.

### 2.6 Immunohistochemistry

Liver sections of 4 μm were deparaffinized and subjected to immunohistochemical analysis of γ‐H2AX and H3K9me3 as described in our previous study.^24^

### 2.7 Oil Red O staining and SA‐β‐gal staining

Liver tissues frozen with OCT compound and cut into 8 μm thickness. LO2 cells were grown in 24‐well plates and the observation of lipid droplets and senescent cells by Oil Red O and SA‐β‐gal staining respectively on the basis of manufacturer's protocol.

### 2.8 Quantitative real‐time polymerase chain reaction (qRT‐PCR)

The total RNA of golden hamster liver was extracted with TRIzol reagent, followed by measurement of its concentration. 2 μg of total RNA was subjected to reverse transcription using the RevertAid First Strand cDNA Synthesis Kit according to the manufacturer' protocol. Glyceraldehyde phosphate dehydrogenase (GAPDH) was used as an irrelevant variable. The mRNA quantification of the target gene was based on the $2^{‐\Delta \Delta C_{T}}$ analysis method and the expression levels were plotted relative to GAPDH mRNA. The primer sequences of related genes were used as follows:

TERT (hamster):

(forward) 5′‐AGGTCAAGAATGCAGGAATGACA‐3′,
(reverse) 5′‐AGTGGTGAGGCTACAATGCC‐3′;

p16 (hamster):

(forward) 5′‐ TGGTCACTGTGAGGATTCAGC‐3′,
(reverse) 5′‐ TGCCCATCATCATCACCTGGTC‐3′;

NCOA4 (hamster).

(forward) 5′‐GGGACCGGAGCCTTTCGT‐3′,
(reverse) 5′‐ TTCATTCTGCCTACTGTTCCAGC‐3′;

GAPDH (hamster):

(forward) 5′‐ GACATCAAGAAGGTGGTGAAGCA‐3′,
(reverse) 5′‐CATCAAAGGTGGAAGAGTGGGA‐3′.

### 2.9 Immunofluorescence staining

Immunofluorescence of liver tissue and LO2 cells were handled as previously reported.^25^ 4', 6‐Diamidino‐2‐phenylindole was used to stain the nucleus of cells. Antifade Mounting Medium was applied to reduce the quenching of fluorescence.

### 2.10 Cell Counting Kit‐8 assay

LO2 cells growing in 96‐well plates were treated by 0.15 mmol/L PA and incubated with different concentration of curcumol for 24 hours. Then, 10 μL of Cell Counting Kit‐8 (CCK‐8) reagent was added to each well and the cells were incubated at 37°C for 2 hours. The absorbance at 450 nm was measured by a Microplate Reader.

### 2.11 Iron content assay

The liver was homogenized immediately in 0.9% saline solution and the supernatant was collected after centrifugation. LO2 cells were lysed with cell lysis solution. The iron content of liver tissue and LO2 cells were measured by relative Iron Content Assay Kit according to the manufacturer’s instructions.

### 2.12 Cell transfection with NCOA4 overexpression plasmid or sh‐YAP

One microgram of NCOA4 overexpression plasmid and 4 μL of UltraCruz® Transfection Reagent were mixed with 150 μL DMEM without FBS at room temperature for 5 minutes respectively. Then, the two mixtures were mixed thoroughly and placed at room temperature for 15 minutes. Cells growing in six‐well plates were rinsed three times with PBS, and the culture medium was replaced with fresh DMEM without FBS. Then, 300 μL of the above mixture was added to each well and the plates were kept shaking while adding. After incubation at 37°C for 6 hours, the culture medium was replaced with a new complete culture medium, and the cells incubated with PA and curcumol for 24 hours. Cell transfection with sh‐YAP is similar to the transfection of NCOA4, except that the transfection reagent is Lipofectamine 2000. The following shRNA sequences of YAP (GenScript) were used:

sh‐YAP#1 (human): 5'‐CACCGAACCAGAGAATCAGTCAGAGTTTCAAGAGAACTCTGACTGATTCTCTGGTTTTTTTTG ‐3';
sh‐YAP#2 (human): 5'‐CACCGAATGTATTGCTGACCTCTTTCTTCAAGAGAGAAAGAGGTCAGCAATACATTTTTTTTG ‐3'.

### 2.13 Western blot assay

LO2 cells and liver tissues were lysed on ice with RIPA lysis buffer for 30 minutes, followed by centrifugation at 15 300 *g* for 15 minutes. Protein concentration of the supernatant was determined by BCA protein assay kit. Western blot analysis was performed as previously described.^26^

### 2.14 Co‐Immunoprecipitation assay

The NCOA4 antibody and the negative control IgG antibody were fixed on the AminoLink coupling resin, respectively. LO2 cells was lysed on ice with IP lysis for 5 minutes and centrifuged for 15 minutes at 13 000 *g*. The samples were taken from the supernatant, and then, the concentration was measured by BCA protein assay kit. An appropriate volume was added to the two resin columns and gently flipped overnight at 4°C. The remaining lysate was used as Input group. The eluate of the resin column was collected, mixed with 5* loading buffer, boiled at 100°C for 5 minutes and then subjected to SDS‐PAGE. The combination of NCOA4 and FTH1 was observed by western blot.

### 2.15 Intracellular ROS levels assay

The intracellular ROS was detected by DCFH‐DA (2,7‐dichlorofluorescein‐diacetate), LO2 cells were cultured in laser confocal dishes and processed as previously described.^22^ Following incubation with DCFH‐DA (1:1000) at 37°C for 20 minutes, cells were washed three times with PBS and pictures were taken with laser confocal microscope.

### 2.16 Statistical analysis

The results were presented as mean ± SEM, and statistical analysis was performed using GraphPad Prism 7.0 (GraphPad Software). The significance of difference was determined by one‐way analysis of variance with the post hoc Dunnett’s test. Values of *P* < .05 were considered statistically significant.

## 3 RESULTS

### 3.1 Curcumol inhibited hepatocyte senescence in golden hamster of NAFLD

**FIGURE 1:**
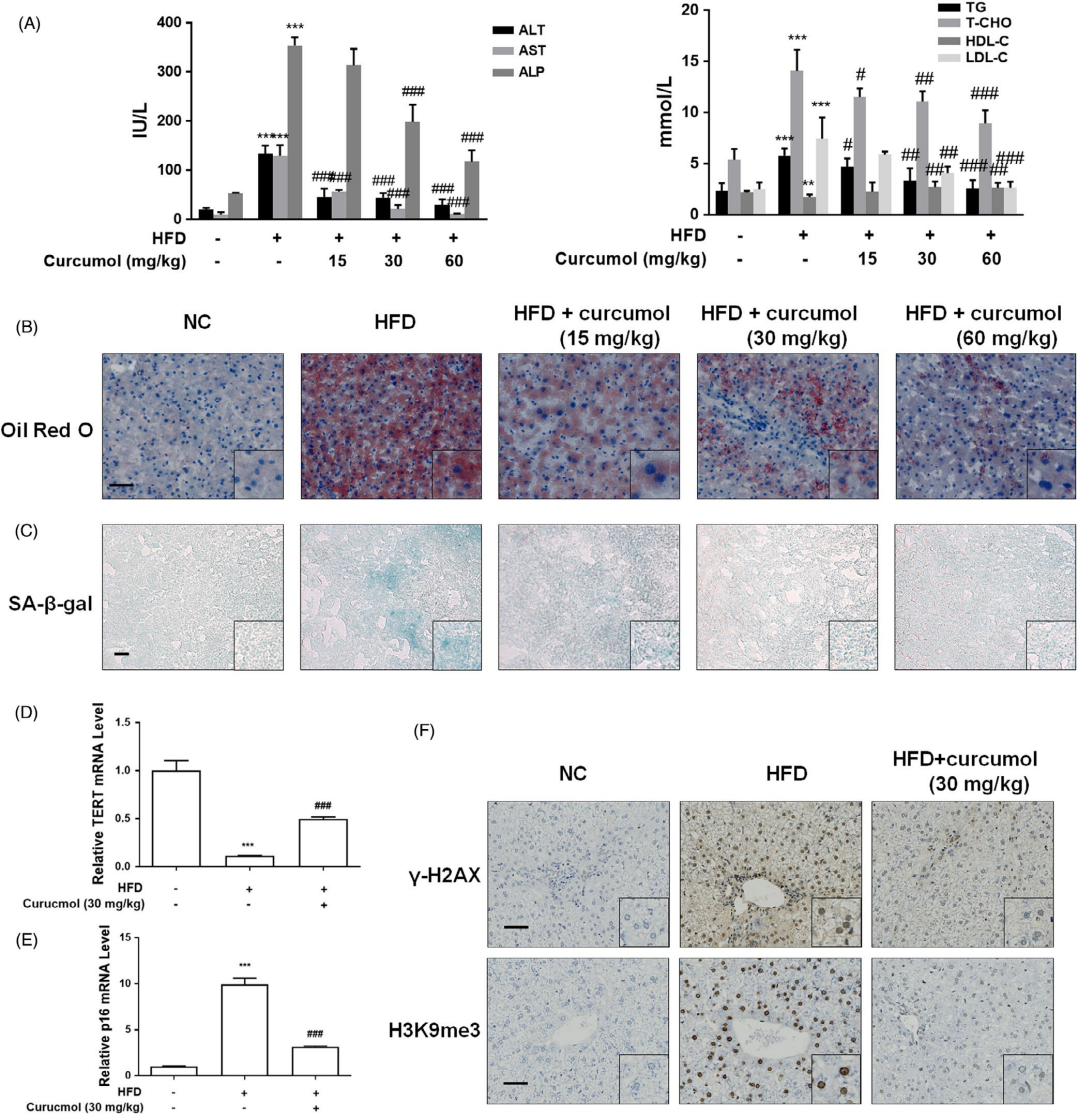
Curcumol inhibited hepatocyte senescence in NAFLD golden hamsters. Male LVG Golden Syrian hamsters were randomly separated into five groups as follows: group 1, vehicle control (Standard diet); group 2, model group (HFD diet); group 3, curcumol‐treated group (15 mg/kg + HFD diet); group 4, curcumol‐treated group (30 mg/kg + HFD diet); group 5, curcumol‐treated group (60 mg/kg + HFD diet). A, Determination of ALT, AST, ALP, TG, T‐CHO, LDL‐C and HDL‐C. Data are represented as mean ± SEM. Significance: ^**^ *P* < .01, ^***^ *P* < .001 vs vehicle control group, ^#^ *P* < .05, ^##^ *P* < .01, ^###^ *P* < .001 vs model group. B,C, Representative microscopic images of Oil Red O and SA‐β‐gal staining. Scale bar, 50 μm (Oil Red O) and 100 μm (SA‐β‐gal). D,E, qRT‐PCR analyses of mRNA expression of TERT and p16. Data are represented as mean ± SEM. Significance: ^***^ *P* < .001 vs vehicle control group, ^###^ *P* < .001 vs model group. F, Representative images of immunohistochemical analysis of γ‐H2AX and H3K9me3 protein in hamster livers. Scale bar, 50 μm

To clarify the mechanism of hepatocyte senescence in NAFLD, we used LVG Golden Syrian hamsters to establish a classic model of NAFLD with HFD. As shown in Figure 1A, compared with the control group, serum levels of ALT, AST, ALP and lipid profiles T‐CHO, TG, LDL‐C were significantly increased, and the level of HDL‐C was obviously decreased in HFD group, indicating that the model of NAFLD has been established. Supplementation with curcumol (15, 30, 60 mg/kg) resulted in a significant amelioration of the mentioned indexes of liver injury and dyslipidemia. The results of Oil Red O staining revealed that there was a large amount of lipid accumulation in the liver tissue of the HFD group, and the lipid droplets were reduced in the liver tissue of curcumol treatment group. When the dose up to 60 mg/kg, the effect of curcumol was even more remarkable (Figure 1B). SA‐β‐gal staining showed that HFD treatment increased the number of senescent hepatocytes, and curcumol could inhibit the senescence of hepatocyte in golden hamster (Figure 1C).

It is well known that one of the biggest characteristics of cellular senescence is the telomere shortening and decreased activity of telomerase. The expression of telomerase reverse transcriptase (TERT) was usually detected to assess telomerase activity.^27^ The analysis of qRT‐PCR showed that curcumol treatment reversed the suppression of TERT activity induced by HFD potently (Figure 1D). In contrast, the effect of curcumol on senescence marker p16 was opposite to TERT (Figure 1E). We then assessed the expression of DNA damage marker γ‐H2AX and heterochromatin marker H3K9me3 by immunohistochemistry staining. The results revealed that the protein levels of γ‐H2AX and H3K9me3 were dramatically increased in the liver of HFD‐fed animals, while in curcumol supplemented groups, the levels of γ‐H2AX and H3K9me3 protein were decreased markedly (Figure 1F). In conclusion, curcumol inhibited the senescence of hepatocyte in golden hamster fed with HFD.

### 3.2 Curcumol reduced cellular senescence in PA‐treated LO2 cells

**FIGURE 2:**
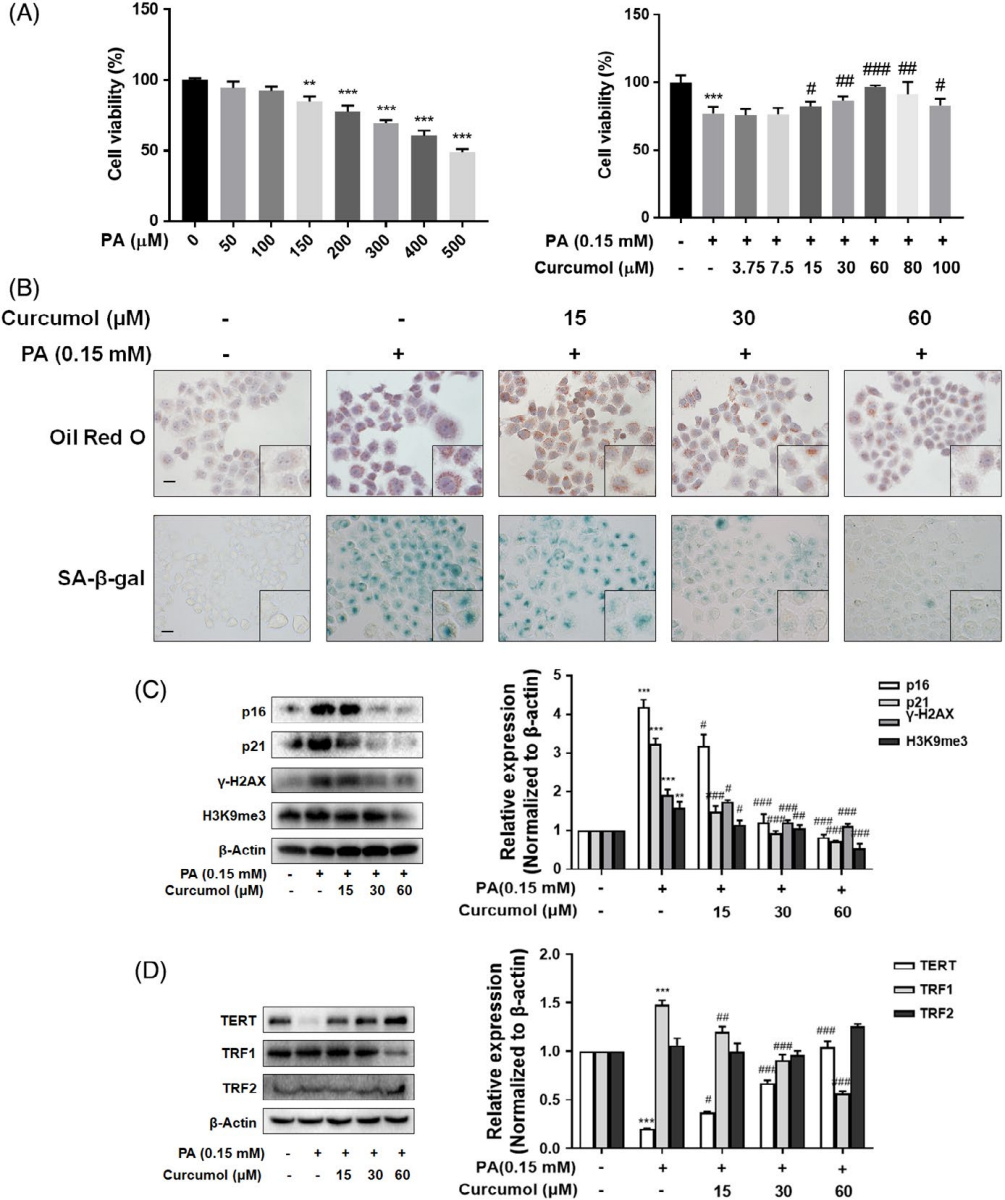
Curcumol reduced cellular senescence in PA‐treated LO2 cells. (A) Cell viability evaluation by Cell Count Kit‐8 analysis. Data are represented as mean ± SEM. Significance: ^**^ *P* < .01, ^***^ *P* < .001 vs vehicle control group, ^#^ *P* < .05, ^##^ *P* < .01, ^###^ *P* < .001 vs PA‐treated group. (B) Representative images of Oil Red O and SA‐β‐gal staining of LO2 cells. Scale bar, 100 μm. C,D, Western blot analyses of the expression of p16, p21, γ‐H2AX, H3K9me3, TERT, TRF1 and TRF2 in LO2 cells. Data are represented as mean ± SEM. Significance: ^**^ *P* < .01, ^***^ *P* < .001 vs vehicle control group, ^#^ *P* < .05, ^##^ *P* < .01, ^###^ *P* < .001 vs PA‐treated group

As shown in Figure 2A, exposure to PA for 24 h induced a marked injury to LO2 cells as assessed by cell viability assay, while pretreatment with curcumol resulted in an evident alleviation of PA‐induced damage. However, when the concentration of curcumol reached 80 μmol/L, the viability of LO2 cells decreased gradually, suggesting that excessively high concentration of curcumol would cause damage to LO2 cells. Therefore, the concentrations of curcumol were set as 15, 30 and 60 μmol/L in subsequent experiments. The results of Oil Red O staining indicated that exposure to PA for 24 hours induced a large number of lipid droplets deposition in the cytoplasm of LO2 cells, and this phenomenon was evidently improved by treatment with curcumol (Figure 2B). Furthermore, the SA‐β‐gal staining assay showed that curcumol was capable of ameliorating PA‐induced cellular senescence in LO2 cells with the levels of senescence‐related markers p16, p21, γ‐H2AX and H3K9me3 decreased significantly (Figure 2C).

When a cell divides, telomeres shorten due to DNA polymerase’s inability to fully replicate the ends of chromosomes, which is termed as the ‘end‐under‐replication problem’.^28^ When telomeres become critically short, cells will activate signalling cascades involving p53 and p21 and start the process of senescence.^29^ TRF1 negatively regulates telomerase‐associated telomere length. TRF2 is associated with stabilizing the telomere structure and also related to telomere shortening.^30^ The result of Western blot displayed that curcumol significantly increased the level of TERT in PA‐treated LO2 cells, indicating that curcumol was able to enhance the activity of telomerase. Similarly, curcumol inhibited PA‐induced expression of TRF1 in LO2 cells but had no significant influence on TRF2 expression (Figure 2D). Collectively, these results inferred that curcumol reduced PA‐induced LO2 senescence in vitro.

### 3.3 Curcumol restrained the senescence of hepatocyte via regulating iron overload

**FIGURE 3:**
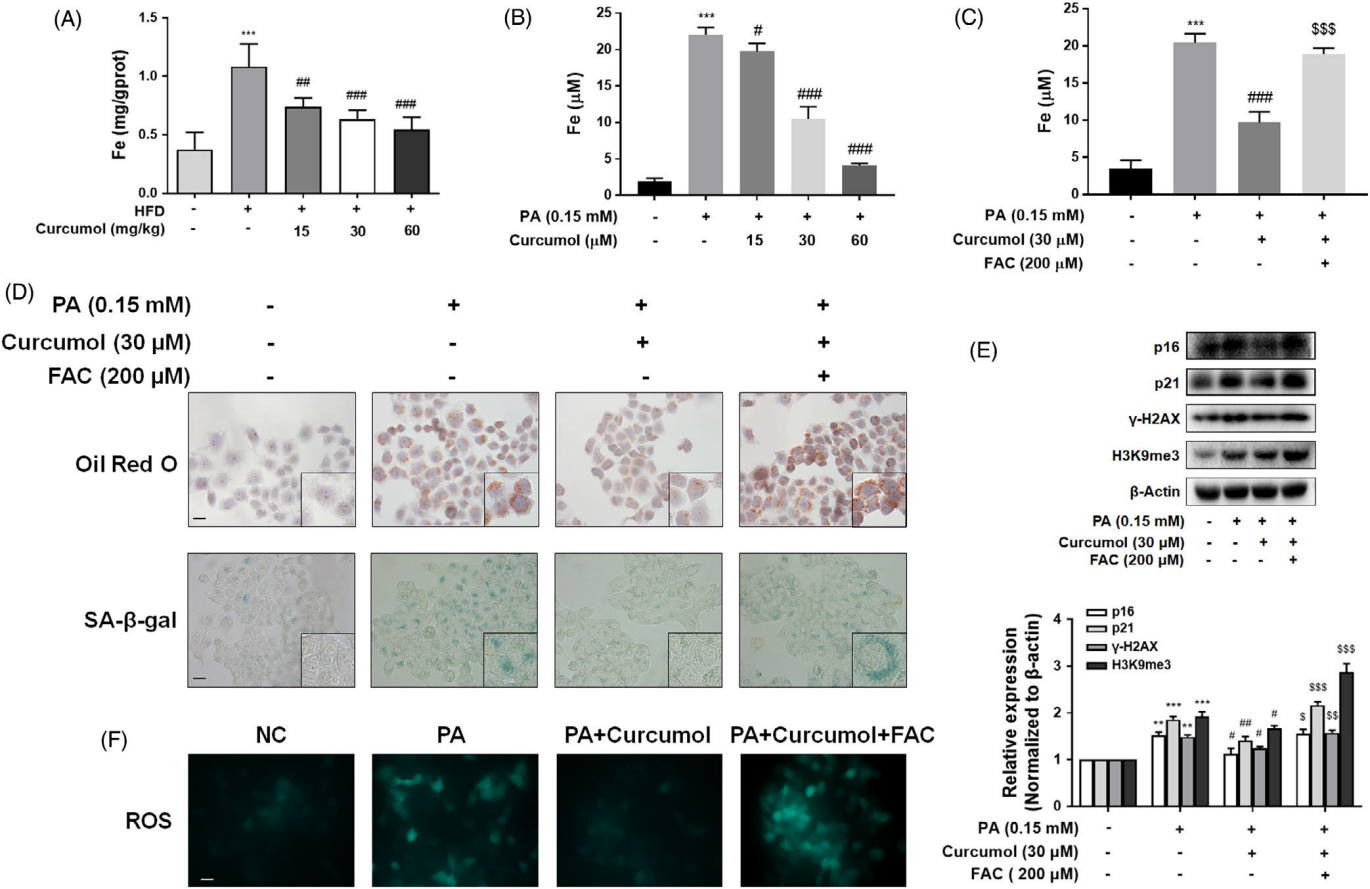
Curcumol restrained the senescence of hepatocyte via regulating iron overload. Determination of iron content in vivo (A) and in vitro (B,C). Data are represented as mean ± SEM. Significance: ^***^ *P* < .001 vs vehicle control, ^#^ *P* < .05, ^##^ *P* < .01, ^###^ *P* < .001 vs PA group, ^$$$^ *P* < .001 vs PA + curcumol (30 μmol/L) group. D, Representative images of Oil Red O and SA‐β‐gal staining of LO2 cells under the indicated conditions. Scale bar, 100 μm. E, Western blot analyses of the protein levels of p16, p21, γ‐H2AX and H3K9me3. Data are represented as mean ± SEM. Significance: ^**^ *P* < .01, ^***^ *P* < .001 vs vehicle control group; ^#^ *P* < .05 ^##^ *P* < .01 vs PA group; ^$^ *P* < .05, ^$$^ *P* < .01, ^$$$^ *P* < .001 vs PA + curcumol (30 μmol/L) group. F, Intracellular ROS content detected by DCFH‐DA staining. Scale bar, 100 μm

To clarify whether the ameliorative effect of curcumol on PA‐induced cellular senescence was derived from alleviation of iron overload, we detected the iron content of NAFLD golden hamster liver samples. The results showed that the iron content was markedly increased in HFD group when compared to the control group, while supplementation with curcumol markedly decreased iron content in the liver of NAFLD hamsters (Figure 3A). In accordance with the in vivo results, the in vitro studies also demonstrated that curcumol could alleviate PA‐induced iron overload in LO2 cells (Figure 3B). To further examine the relationship between iron overload and cellular senescence, LO2 cells were treated with FAC (an inducer of iron overload) before exposure to PA. The results showed that preincubation with FAC could weaken the effect of curcumol on iron content in PA‐treated LO2 cells (Figure 3C), and interfere with curcumol’s therapeutic efficacy on lipid accumulation and cellular senescence (Figure 3D). Similarly, FAC abolished the inhibitory effect of curcumol on the levels of senescence markers (Figure 3E). In order to confirm that the overproduction of ROS played an important role in iron overload regulation of hepatocyte senescence, DCFH‐DA was used to detect the ROS generation in the presence or absence of FAC. Observation using fluorescence microscopy displayed that curcumol reduced ROS in PA‐treated LO2 cells, whereas the effect of curcumol on ROS level was weakened by FAC (Figure 3F). Taken together, curcumol restrained the senescence of hepatocyte via inhibiting iron overload in PA‐treated LO2 cells.

### 3.4 Curcumol suppressed ferritinophagy through inhibition of NCOA4

**FIGURE 4:**
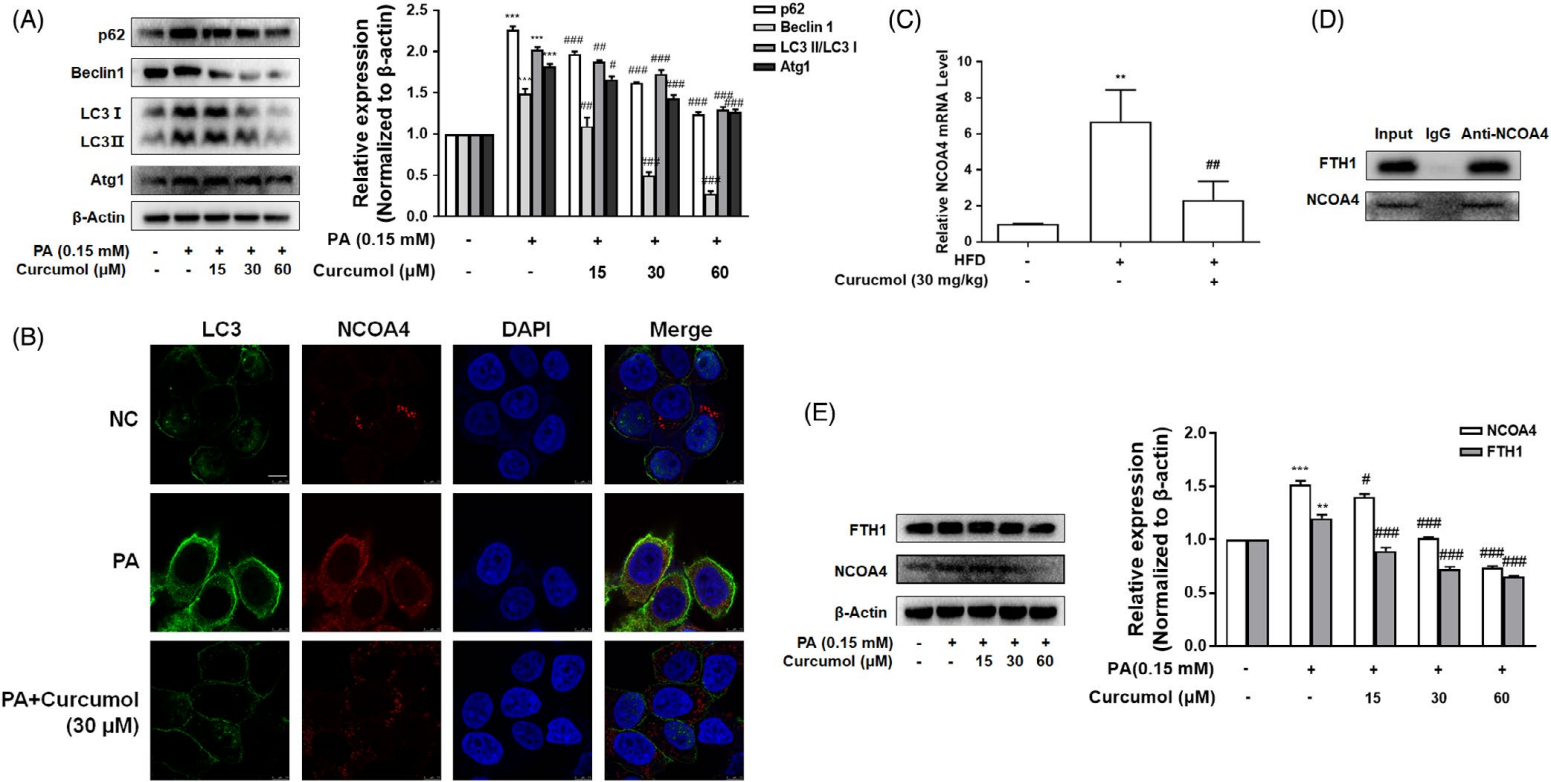
Curcumol suppressed ferritinophagy through inhibition of NCOA4. A, Western blot analyses of the protein levels of p62, Beclin1, LC3Ⅱ/ LC3Ⅰ and Atg1 proteins. Data are represented as mean ± SEM. Significance: ^***^ *P* < .001 vs vehicle control group; ^#^ *P* < .05, ^##^ *P* < .01, ^###^ *P* < .001 vs PA group. B, Immunofluorescence staining analysis of the interaction of NCOA4 and LC3. Scale bar, 7.5 μm. C, qRT‐PCR analyses of NCOA4 mRNA expression in vivo. Data are represented as mean ± SEM. Significance: ^**^ *P* < .01 vs NC group; ^##^ *P* < .01 vs HFD group. D, Co‐IP analysis of the relevance between NCOA4 and FTH1. E, Western blot analyses of the protein expression of NCOA4 and FTH1 in vitro. Data are represented as mean ± SEM. Significance: ^**^ *P* < .01, ^***^ *P* < .001 vs vehicle control group; ^#^ *P* < .05, ^###^ *P* < .001 vs PA group

To validate the effect of ferritinophagy on the regulation of iron overload, we tested the levels of p62, Beclin1, LC3Ⅱ/LC3Ⅰ and Atg1, the key indicators of autophagy pathway. As shown in Figure 4A, the levels of these factors were upregulated in PA‐treated LO2 cells and downregulated by curcumol. NCOA4, a specific mediator of ferritinophagy, played a critical role in selective degradation of ferritin. The results of immunofluorescence microscopy indicated that, in PA‐treated LO2 cells, NCOA4 mainly accumulates in autophagosomes, of which LC3 was used as a classic marker. Curcumol could suppress the formation of autophagosomes and decrease the accumulation of NCOA4 in autophagosome (Figure 4B). Consistent with the in vitro results, curcumol effectively reversed the upregulation of NCOA4 mRNA in livers of hamsters fed with HFD (Figure 4C). Based on the above results, it is supposed that NCOA4 targets the subunit of ferritin heavy chain (FTH1) to induce iron overload. The results of Co‐IP showed that NCOA4 interacts with FTH1 in PA‐treated LO2 cells (Figure 4D), and curcumol could inhibit the effect of PA on the expression of NCOA4 (Figure 4E). Besides, curcumol treatment attenuated the effect of PA on the level of FTH1 as well (Figure 4E). In summary, these results suggested that NCOA4 accumulated in autophagosomes combined with FTH1 and promoted the release of iron, and curcumol could reduce PA‐induced iron release through inhibition of NCOA4 in LO2 cells.

### 3.5 Overexpression of NCOA4 weakened the effect of curcumol on cellular senescence in vitro

**FIGURE 5:**
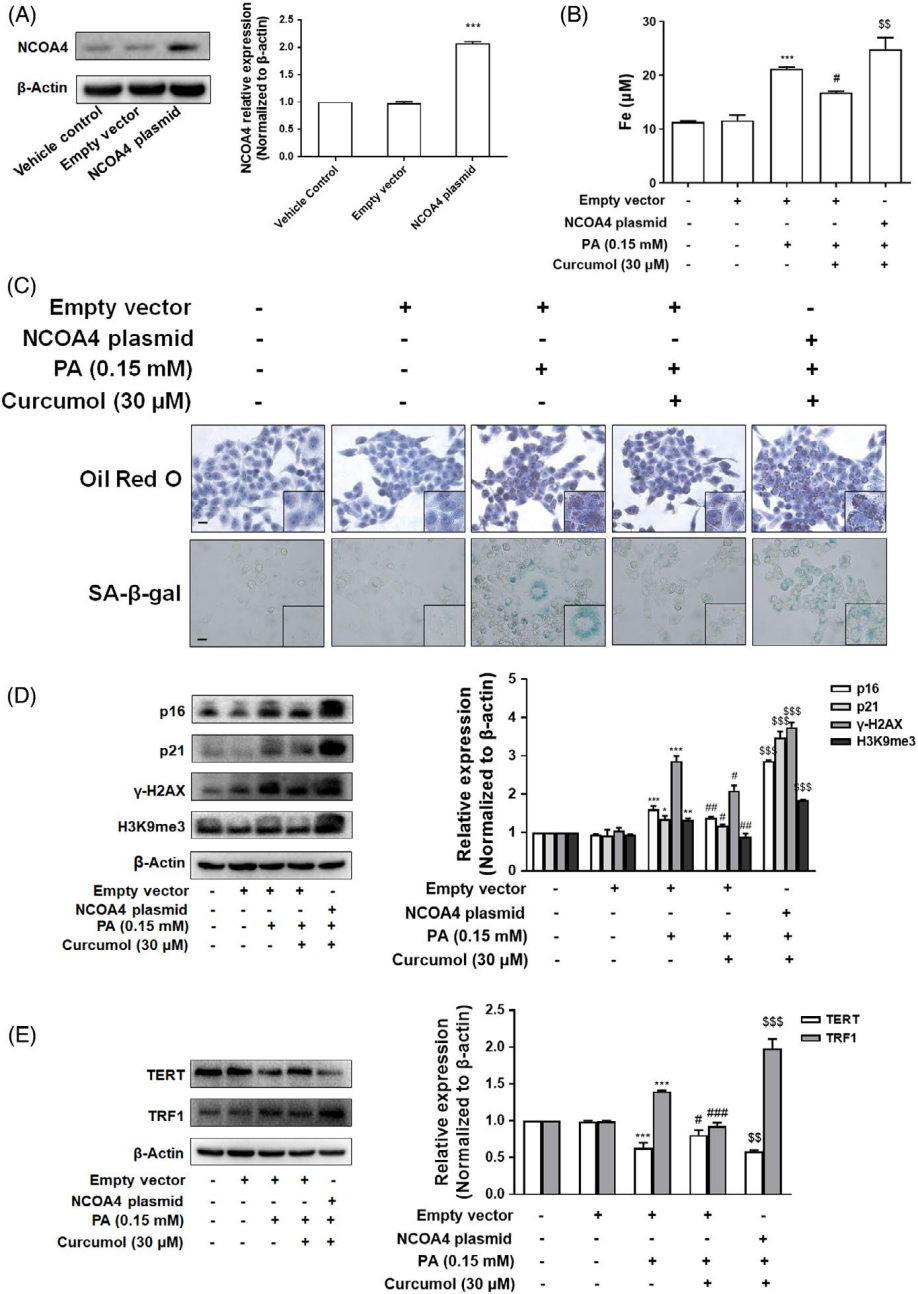
Overexpression of NCOA4 weakened the effect of curcumol on cellular senescence in PA‐treated LO2 cells. A, The transfection efficiency of NCOA4 plasmid measured by western blot analysis. Data are represented as mean ± SEM. Significance: ^***^ *P* < .001 vs Empty vector group. B, Iron content assessed by Iron Colorimetric Assay Kit. Data are represented as mean ± SEM. Significance: ^***^ *P* < .001 vs Empty vector group, ^#^ *P* < .05 vs Empty vector + PA group, ^$$^ *P* < .01 vs Empty vector + PA + curcumol (30 μmol/L) group. C, Representative images of Oil Red O and SA‐β‐gal staining of LO2 cells. Scale bar, 100 μm. D,E, Western blot analyses of the protein levels of p16, p21, γ‐H2AX, H3K9me3, TERT and TRF1 in LO2 cells. Data are represented as mean ± SEM. Significance: ^*^ *P* < .05, ^**^ *P* < .01, ^***^ *P* < .001 vs Empty vector group, ^#^ *P* < .05, ^##^ *P* < .01, ^###^ *P* < .001 vs Empty vector + PA group; ^$$^ *P* < .01, ^$$$^ *P* < .001 vs Empty vector + PA + curcumol (30 μmol/L) group

To explore whether curcumol reduces hepatocyte senescence through inhibition of NCOA4, LO2 cells were transfected with NCOA4 CRISPR Activation Plasmid and the transfection efficiency was confirmed by Western blot (Figure 5A). At first, the effect of curcumol on iron deposition was reversed by overexpression of NCOA4 (Figure 5B). Then, we assessed the role of NCOA4 on lipid accumulation and cellular senescence by Oil Red O and SA‐β‐gal staining. The results showed that the effects of curcumol on lipid accumulation and cellular senescence were attenuated by transfection with NCOA4 plasmid (Figure 5C). Besides, overexpression of NCOA4 weakened the effect of curcumol on the expression of cellular senescence‐related markers p16, p21, γ‐H2AX, H3K9me3 (Figure 5D). Similar observations were found in the indices of telomere and telomerase system TERT and TRF1 (Figure 5E).

### 3.6 NCOA4 impaired curcumol's inhibition of hepatocyte senescence in vivo

**FIGURE 6:**
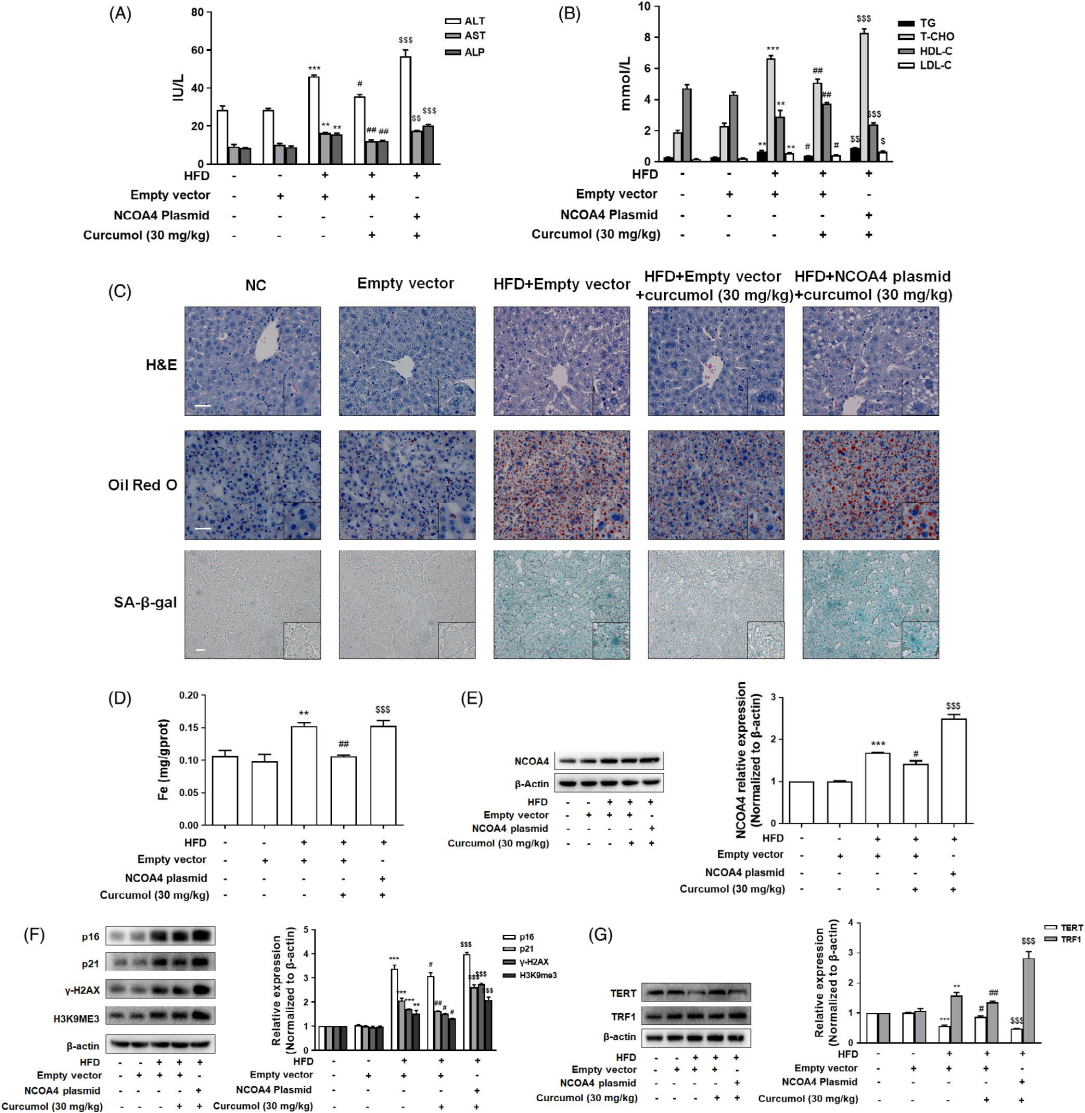
NCOA4 impaired curcumol’ inhibition of hepatocyte senescence in vivo. A,B, Serum levels of ALT, AST, ALP, TG, T‐CHO, HDL‐C and LDL‐C in golden hamsters. Data are represented as mean ± SEM. Significance: ^**^ *P* < .01, ^***^ *P* < .001 vs Empty vector group; ^#^ *P* < .05, ^##^ *P* < .01 vs HFD + Empty vector group; ^$^ *P* < .05, ^$$^ *P* < .01, ^$$$^ *P* < .001 vs HFD + Empty vector + Curcumol (30 mg/kg) group. C, Representative photographs of liver sections stained with H&E, Oil Red O and SA‐β‐gal staining. Scale bar, 50 μm (H&E, Oil Red O), 100 μm (SA‐β‐gal). D, The iron content of liver evaluated by Tissue Iron Colorimetric Assay Kit. Data are represented as mean ± SEM. Significance: ^**^ *P* < .01 vs Empty vector; ^##^ *P* < .01 vs HFD + Empty vector group; ^$$$^ *P* < .001 vs HFD + Empty vector + Curcumol (30 mg/kg) group. E‐G, Western blot analyses of the transfection efficiency of adenovirus‐mediated NCOA4 plasmid and the protein levels of p16, p21, γ‐H2AX, H3K9me3, TERT and TRF1 in liver tissues. Data are represented as mean ± SEM. Significance: ^**^ *P* < .01, ^***^ *P* < .001 vs Empty vector group; ^#^ *P* < .05, ^##^ *P* < .01 vs HFD + Empty vector group; ^$$$^ *P* < .001 vs HFD + Empty vector + Curcumol (30 mg/kg) group

To further determine the exact role of NCOA4 in regulating iron overload and cellular senescence in vivo, C57BL/6J mice were transfected with lentivirus‐packaged NCOA4 plasmid followed by HFD treatment. The results demonstrated that overexpression of NCOA4 in HFD fed mice attenuated the ameliorative effect of curcumol against hepatocellular damage as indicated by serum levels of ALT, AST and ALP (Figure 6A). Moreover, NCOA4 plasmid weakened the regulatory effect of curcumol on the levels of TG, T‐CHO, HDL‐C and LDL‐C (Figure 6B). H&E, Oil Red O and SA‐β‐gal staining showed that the effects of curcumol on hepatic damage, lipid accumulation and hepatocyte senescence were attenuated in the mice transfected with NCOA4 plasmid (Figure 6C). In addition, we assessed the effect of NCOA4 on curcumol’s inhibition of iron overload. We found that overexpression of NCOA4 weakened the inhibitory effect of curcumol on iron deposition (Figure 6D). Furthermore, the suppressive effects of curcumol on senescence markers and telomerase system indices were reversed by NCOA4 plasmid transfection (Figure 6E‐G). Overall, these data indicated that NCOA4 overexpression neutralized the inhibitory effect of curcumol on hepatocyte senescence in vivo.

### 3.7 Curcumol inhibited NCOA4 expression via a YAP‐dependent mechanism

**FIGURE 7:**
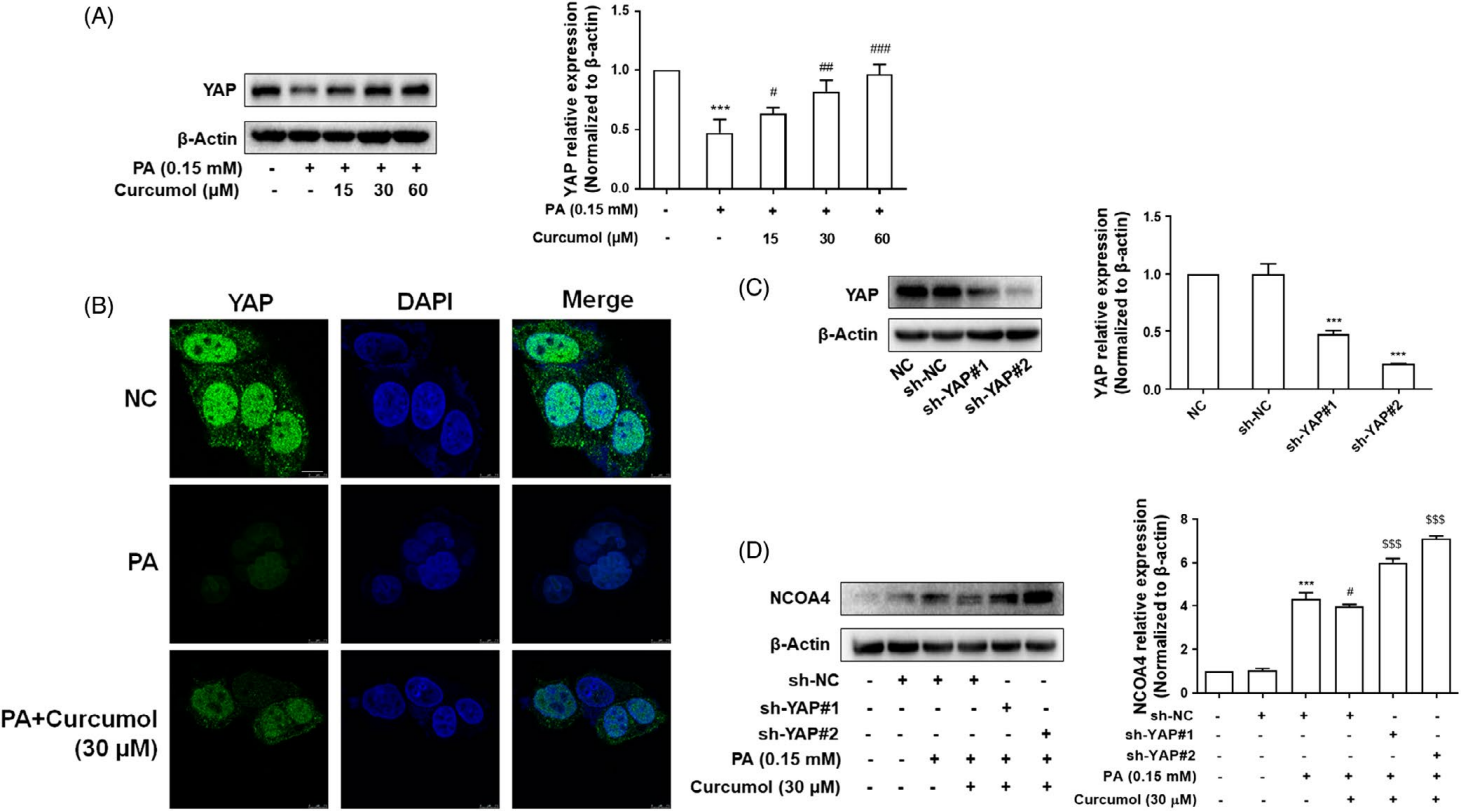
Curcumol inhibited the NCOA4 expression via a YAP‐dependent mechanism. A,B, Western blot and Immunofluorescence staining analyses of the expression of YAP. Scale bar, 7.5 μm. Data are represented as mean ± SEM. Significance: ^***^ *P* < .001 vs vehicle control group; ^#^ *P* < .05, ^##^ *P* < .01, ^###^ *P* < .001 vs PA‐treated group. C, Western blot analysis evaluated the transfection efficiency of YAP under the treatment of sh‐YAP#1 and sh‐YAP#2 plasmid. Data are represented as mean ± SEM. Significance: ^***^ *P* < .001 vs sh‐NC group. D, Western blot analysis of the expression of NCOA4. Data are represented as mean ± SEM. Significance: ^***^ *P* < .001 vs sh‐NC group, ^#^ *P* < .05 vs sh‐NC+PA group; ^$$$^ *P* < .001 vs sh‐NC + PA + Curcumol (30 μmol/L) group

**FIGURE 8:**
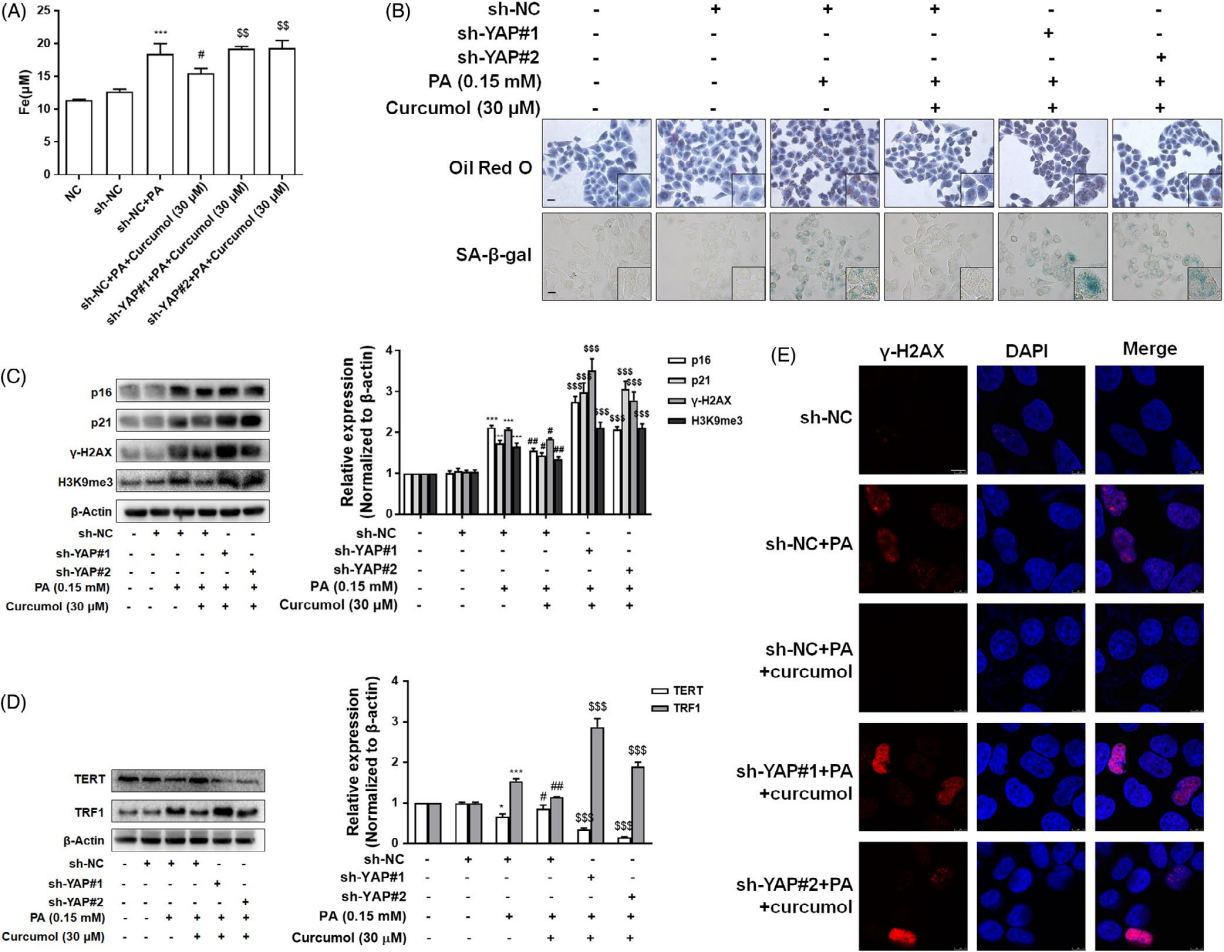
Curcumol suppressed the senescence of hepatocyte through promotion of YAP. A, Iron content in LO2 cells evaluated by Iron Content Assay Kit. Data are represented as mean ± SEM. Significance: ^***^ *P* < .001 vs sh‐NC group; ^#^ *P* < .05 vs sh‐NC + PA group; ^$$^ *P* < .01 vs sh‐NC + PA + curcumol (30 μmol/L) group. B, Representative images of Oil Red O and SA‐β‐gal staining of LO2 cells under the indicated conditions. Scale bar, 100 μm. C,D, Western blot analyses of the protein level of p16, p21, γ‐H2AX, H3K9me3, TERT, TRF1 in LO2 cells. Data are represented as mean ± SEM. Significance: ^*^ *P* < .05, ^**^ *P* < .01, ^***^ *P* < .001 vs sh‐NC group; ^#^ *P* < .05, ^##^ *P* < .01, ^###^ *P* < .001 vs sh‐NC + PA group; ^$$$^ *P* < .001 vs sh‐NC +PA + curcumol (30 μmol/L) group. E, Immunofluorescence staining analyses of γ‐H2AX. Scale bar, 7.5 μm

It is well known that the formation of autophagosomes is an important part of ferritinophagy. YAP, a major downstream effector of the Hippo signalling pathway, may affect the formation of autophagosomes^18^ and cellular senescence.^31^, ^32^ It is unclear whether the inhibition of curcumol on hepatocyte senescence is related to its regulation of YAP and subsequent suppression of ferritinophagy and iron overload. Firstly, the analyses of Western blot and immunofluorescence showed that curcumol treatment markedly increased the protein level of YAP in vitro (Figure 7A,B). As shown in Figure 7C,D, inhibition of YAP expression with YAP shRNA (sh‐YAP) could weaken the effect of curcumol on the level of NCOA4 in PA‐treated cells. To gain further insight into the relationship between YAP and iron overload, we detected the iron content and found that the effect of curcumol on iron overload was attenuated by sh‐YAP (Figure 8A). Moreover, we found that the effects of curcumol on lipid accumulation and cellular senescence were reversed by sh‐YAP (Figure 8B). Figure 8C,D showed that the suppression of YAP could weaken the effects of curcumol on the expression of senescence‐related markers, telomere and telomerase system indices. Furthermore, the immunofluorescence analysis showed that γ‐H2AX positive cells were significantly increased in sh‐YAP treated LO2 cells when compared to curcumol‐treated group (Figure 8E). Taken together, these findings indicated that curcumol inhibited NCOA4 expression and cellular senescence via a YAP‐dependent mechanism.

## 4 DISCUSSION

The current study provided the first evidence that inhibition of hepatocyte senescence by curcumol would be beneficial to the treatment of NAFLD. In the current report, supplementation with curcumol improved liver damage and hepatic steatosis in HFD‐fed golden hamsters. Besides, curcumol inhibited HFD‐induced hepatocyte senescence by the detection of SA‐β‐Gal, p16, telomerase activity index TERT, heterochromatin marker H3K9me3 and DNA damage marker γ‐H2AX in vivo. To further investigate the mechanism of curcumol on cellular senescence, we treated LO2 cells with PA to construct a cell model of lipid metabolism disorders. It was found that the optimal concentration of curcumol on 15‐60 μmol/L could significantly improve the cell viability in PA‐treated LO2 cells. Curcumol inhibited PA‐induced lipid accumulation and cellular senescence in LO2 cells. Moreover, curcumol could reverse the effect of PA on cellular senescence by the examination of age‐related indicators p16, p21, γ‐H2AX, H3K9me3, telomere and telomerase system index TERT and TRF1. However, we found curcumol has no significant effect on the expression of TRF2. This result suggested that the effect of curcumol on telomere and telomerase system mainly by regulating the levels of TERT and TRF1. In sum, these findings showed that curcumol could suppress the cellular senescence in vivo and in vitro.

Some studies have shown that iron overload could induce ROS accumulation and then lead to cell dysfunction, such as cellular senescence.^17^, ^33^ The liver is the main iron storage organ and the excessive accumulation of iron in hepatocytes leads to the pathological iron overload, which could promote the production of ROS and affect the normal function of hepatocyte. These researches provide us with an idea whether curcumol can affect hepatocyte senescence by regulating iron overload in NAFLD models. In this work, the iron content was elevated in HFD‐fed hamster or PA‐treated LO2 cells and curcumol could reverse these effects. Then, we used iron overload inducer FAC to explore whether curcumol reduces hepatocyte senescence by inhibiting pathological iron overload. The result showed that FAC partly recover the reduced iron content of curcumol in PA‐treated LO2 cells. Moreover, the inhibitory effects of curcumol on lipid accumulation and cellular senescence could be reversed by FAC. In addition, FAC attenuated the effect of curcumol on the level of ROS. As substantial evidence, curcumol treatment decreased iron overload in PA‐damaged LO2 cells and this effect certainly contributes to the inhibition of cellular senescence. However, the important role of ROS on curcumol regulating iron overload and inhibiting hepatocyte senescence has not been comprehensively studied.

NCOA4, a selective cargo receptor for autophagic turnover of ferritin (ferritinophagy), is critical for iron homeostasis.^14^ A key surface arginine in FTH1 direct association with a C‐terminal element in NCOA4 is required for delivery of ferritin to the lysosome via autophagosomes.^15^ Considering that NCOA4 has been implicated to critically determine the development and progression of ferritinophagy associated with iron overload, it is necessary to address how curcumol regulation of NCOA4 alters iron overload. In the present study, we first confirmed that curcumol suppressed the levels of autophagosome formation marker LC3, Atg1, Beclin 1, which were elevated by PA in LO2 cells. Moreover, we demonstrated that the formation of autophagy was accompanied by the increase of NCOA4 in PA treatment. Conversely, curcumol in PA‐treated LO2 cells led to a significant inhibition of this phenomenon. In addition, our data revealed that curcumol inhibited the effect of PA on the interaction between NCOA4 and FTH1. To explore whether curcumol regulates pathological iron overload by regulating NCOA4, we detected the effect of NCOA4 plasmid on the iron content in curcumol‐treated LO2 cells. Our data revealed that NCOA4 overexpression could weaken the effect of curcumol on iron content. Furthermore, we investigated the effect of NCOA4 plasmid on curcumol regulation of lipid accumulation and cellular senescence. The inhibition of curcumol on cellular senescence and lipid droplet deposition was largely aggravated by transfection of NCOA4 plasmid in PA‐treated LO2 cells. These findings verified that the inhibitory effect of curcumol on pathological iron overload would help to the reduction of senescent cell by regulating NCOA4/ferritinophagy. To further support the notion that overexpression of NCOA4 accounted for aggravation of steatohepatitis and metabolic deterioration in C57BL/6J mice and could override any beneficial effects of curcumol. We exogenously administered a lentivirus‐packaged NCOA4 plasmid into mice through the tail vein. The induction of NCOA4 on hepatic lipid accumulation and cellular senescence that ultimately weakened the effects of curcumol. These in vivo results are consistent with the above findings.

The negative regulation of YAP on the formation of autophagosome was previously reported in several cancer cell types.^34^ However, there is no evidence showed that YAP could regulate ferritinophagy. It is further found that YAP played an important role in the cellular senescence. Based on these findings, we postulated and verified that the stimulation of YAP by curcumol is involved in the effect of curcumol on suppression of NCOA4. In support of this, we found that curcumol up‐regulated the protein expression of YAP firstly in PA‐treated LO2 cells. Then, we validated that sh‐YAP weakened the effect of curcumol on NCOA4 expression. In addition, we found that disruption of YAP impaired the effects of curcumol on lipid deposition and cellular senescence in PA‐stimulated LO2 cells. Although the underlying mechanism on YAP regulation of NCOA4 require further investigation, targeting YAP using curcumol may become a potential therapeutic strategy for NAFLD.

## 5 CONCLUSION

**FIGURE 9:**
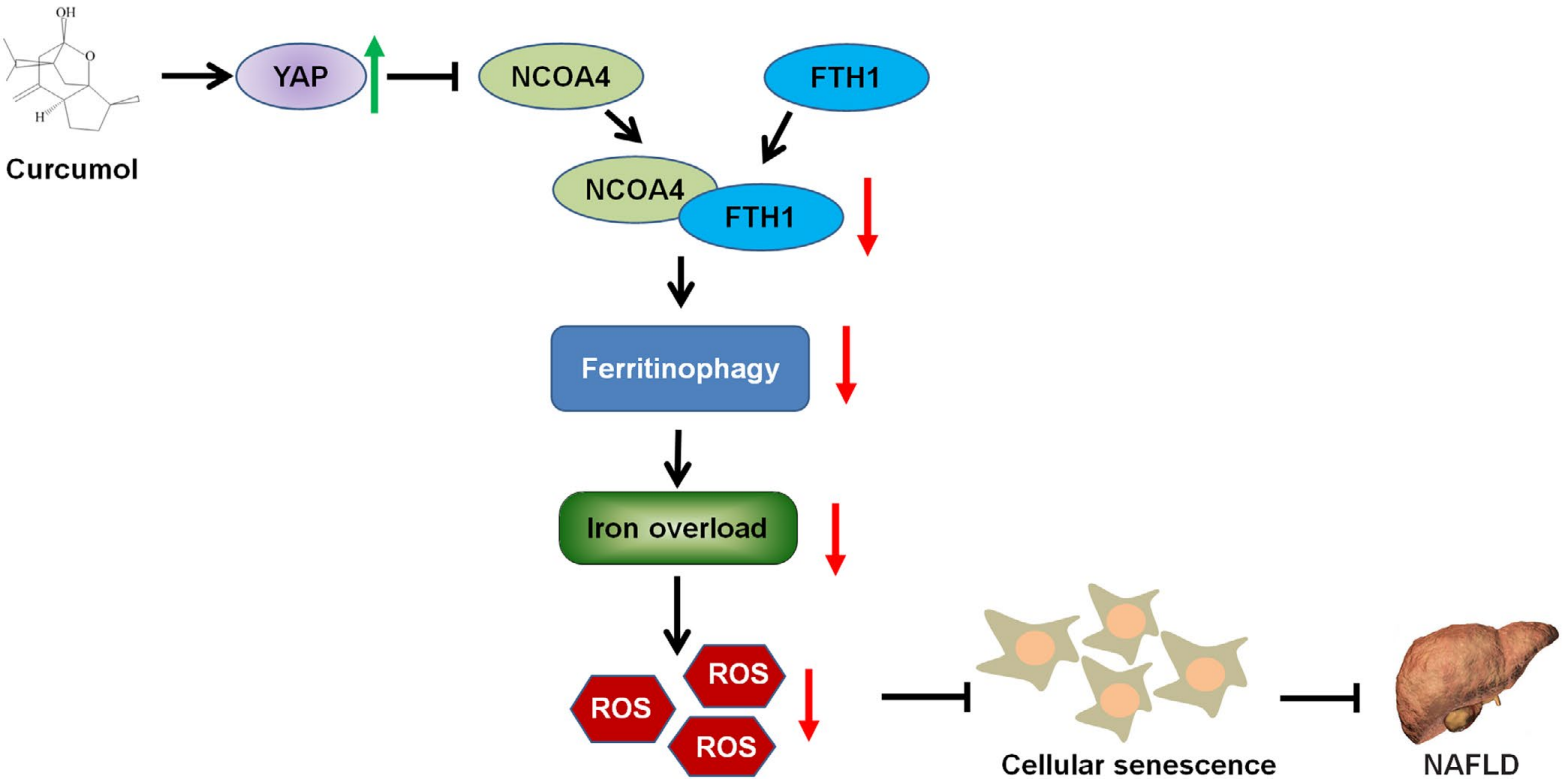
Curcumol inhibited hepatocyte senescence by suppressing ferritinophagy‐mediated iron overload. Furthermore, this effect of curcumol is related to the regulation of YAP/NCOA4 in non‐alcoholic fatty liver disease (NAFLD) models

In summary, the collected data have exhibited that curcumol protected hepatocyte from lipid accumulation and cellular senescence by regulating YAP/NCOA4 in NAFLD (Figure 9). However, more work is needed to be undertaken to clarify the definite mechanism on YAP regulation of NCOA4 by curcumol. This study is helpful for curcumol to become a novel effective natural compound for the prevention and improvement of NAFLD.

## CONFLICT OF INTEREST

Authors have declared that they have no conflicts of interest to this study.

## AUTHOR CONTRIBUTIONS

Huanhuan Jin and Shuguo Zheng conceived and designed this study. Xiaoyu Qi, Anping Song, Mingyue Ma, Peipei Wang and Xinbei Zhang performed the experiments in vivo and in vitro. Huanhuan Jin and Xiaoyu Qi analysed the data. Chunfeng Lu and Junxiu Zhang reviewed and revised the manuscript. All authors read and approved the final manuscript for publication.

## Data Availability

The data that support the findings of this study are available from the corresponding author upon reasonable request.
